# Pulmonary endarterectomy normalizes interventricular dyssynchrony and right ventricular systolic wall stress

**DOI:** 10.1186/1532-429X-14-5

**Published:** 2012-01-12

**Authors:** Gert-Jan Mauritz, Anton Vonk-Noordegraaf, Taco Kind, Sulaiman Surie, Jaap J Kloek, Paul Bresser, Nabil Saouti, Joachim Bosboom, Nico Westerhof, J Tim Marcus

**Affiliations:** 1 Department of Pulmonary Diseases, University of Amsterdam, Amsterdam, The Netherlands; 2 Department of Physics and Medical Technology, University of Amsterdam, Amsterdam, The Netherlands; 3 Department of Physiology, Institute for Cardiovascular Research, VU University Medical Center, Amsterdam, the Netherlands; 4 Department of Pulmonary Diseases, University of Amsterdam, Amsterdam, The Netherlands; 5 Department of Cardiothoracic Surgery of the Academic Medical Center, University of Amsterdam, Amsterdam, The Netherlands; 6 Department of Respiratory Medicine, Onze Lieve Vrouwe Gasthuis, Amsterdam, the Netherlands

**Keywords:** Chronic Thrombo-Embolic Pulmonary Hypertension, Pulmonary Endarterectomy, interventricular mechanical asynchrony, myocardial strain, wall stress

## Abstract

**Background:**

Interventricular mechanical dyssynchrony is a characteristic of pulmonary hypertension. We studied the role of right ventricular (RV) wall stress in the recovery of interventricular dyssynchrony, after pulmonary endarterectomy (PEA) in chronic thromboembolic pulmonary hypertension (CTEPH).

**Methods:**

In 13 consecutive patients with CTEPH, before and 6 months after pulmonary endarterectomy, cardiovascular magnetic resonance myocardial tagging was applied. For the left ventricular (LV) and RV free walls, the time to peak (Tpeak) of circumferential shortening (strain) was calculated. Pulmonary Artery Pressure (PAP) was measured by right heart catheterization within 48 hours of PEA. Then the RV free wall systolic wall stress was calculated by the Laplace law.

**Results:**

After PEA, the left to right free wall delay (L-R delay) in Tpeak strain decreased from 97 ± 49 ms to -4 ± 51 ms (*P *< 0.001), which was not different from normal reference values of -35 ± 10 ms (*P *= 0.18). The RV wall stress decreased significantly from 15.2 ± 6.4 kPa to 5.7 ± 3.4 kPa (*P *< 0.001), which was not different from normal reference values of 5.3 ± 1.39 kPa (*P *= 0.78). The reduction of L-R delay in Tpeak was more strongly associated with the reduction in RV wall stress (r = 0.69,*P *= 0.007) than with the reduction in systolic PAP (r = 0.53, *P *= 0.07). The reduction of L-R delay in Tpeak was not associated with estimates of the reduction in RV radius (r = 0.37,*P *= 0.21) or increase in RV systolic wall thickness (r = 0.19,*P *= 0.53).

**Conclusion:**

After PEA for CTEPH, the RV and LV peak strains are resynchronized. The reduction in systolic RV wall stress plays a key role in this resynchronization.

## Background

Interventricular mechanical dyssynchrony is a characteristic of right ventricular (RV) pressure overload [1-3]. It occurs at the end of RV myocardial shortening, when the RV free wall continues shortening while the left ventricular (LV) wall is already in its early diastolic phase [4-7]. Consequently, the ventricular septum bows to the left, and the RV shortens without ejection thereby making the RV very inefficient [8], and in addition impairing early LV filling [9,10]. The underlying mechanism of this prolonged RV contraction duration is unknown. In an isolated Langendorf-perfused heart, Handoko et al [11] created a L-R dyssynchrony in peak pressure by increasing RV pressure using inflation of a balloon. Two earlier studies have found a relation between L-R dyssynchrony and wall stress [5,12]. Since wall stress is the combined effect of pressure, volume and wall thickness, the questions remains whether dyssynchrony is best explained by RV pressure or the combination of the variables as expressed by wall stress. This insight is relevant for a better understanding of the adaptation mechanisms of RV structure in the presence of right ventricular overload.

The aim of the present study is twofold. First to assess the effect of RV unloading on dyssynchrony in Chronic Thrombo-Embolic Pulmonary Hypertension (CTEPH). Secondly, to separate the effects on dyssynchrony induced by pressure, volume, wall thickness and wall stress. We assessed these effects on L-R dyssynchrony in CTEPH patients, before and after pulmonary endarterectomy.

## Methods

### Patients population

Thirteen of 17 consecutive patients with surgically accessible CTEPH, referred to the Academic Medical Center of the University of Amsterdam, were prospectively studied before and after pulmonary endarterectomy (PEA). One patient refused to participate because of claustrophobia, 1 patient died postoperatively, and 2 patients refused to undergo a second cardiovascular magnetic resonance (CMR) after surgery. Diagnosis of CTEPH and eligibility for PEA were established on the basis of previously reported procedures and criteria [13]. Diagnosis and cardiopulmonary hemodynamics were determined by pulmonary angiography and right heart catheterization. Coronary angiography was routinely performed in all patients older than 50 years of age, and in patients older than 40 years of age if they had a history of smoking.

In addition, eight healthy subjects (called 'control') were included (age 55 ± 6 years, 3 women), with normal electrocardiogram (ECG) and QRS width of 80 ± 12 ms, where RV and LV wall strains were obtained and compared with the CTEPH group. For the estimation of normal RV wall stress, we included also 8 patients (called 'normal right-sided pressure group') suspected of having PH (age 59 ± 11 years, 5 women) but with normal right-sided pressures confirmed by right heart catheterisation. All patients and controls gave informed consent to the study protocol, which was approved by the institutional review board of the VU University Medical Center.

### Right heart catheterization

All patients underwent right heart catheterization within 48 h of their pre-operative CMR. Right heart catheterisation gave right atrial pressure, pulmonary artery pressure (PAP), pulmonary capillary wedge pressure (PCWP), and cardiac output (thermodilution). Pulmonary vascular resistance (PVR) was calculated as: PVR = 80 · (mean PAP-mean PCWP)/cardiac output. Postoperative hemodynamic measurements were repeated on the first or second day following PEA, before removal of the Swan-Ganz catheter (Edwards LifeSciences, Irvine, CA, USA)

### CMR Imaging acquisition

All patients underwent CMR myocardial tagging at baseline before, and at least 6 months after endarterectomy. A 1.5 Siemens 'Avanto' whole body MRI system, equipped with a 6-element phased-array coil was used (Siemens Medical Solutions, Erlangen, Germany). CMR myocardial tagging with high temporal resolution (29 ms) was applied with Complementary Spatial Modulation of Magnetization (7 mm tag distance) and steady state free precession imaging. Parameters: Eight phase-encoding lines per heart beat, TR 3.6 ms, TE 1.8 ms, flip angle 20 deg, voxel size 1.2 × 3.8 × 6.0 mm3. In all patients and control subjects this tagging cine was acquired in the mid-ventricular short-axis plane. After the tagging acquisitions, the LV and RV were covered by a stack of short-axis cine CMR images for volumetric assessment, using steady state free precession imaging with a temporal resolution between 25 and 35 ms.

### CMR Image analysis

End-diastolic volume (EDV), end-systolic volume (ESV), ejection fraction and myocardial mass were calculated using MR Analytical Software System (Medis, Leiden, The Netherlands). In order to assess LV peak filling rate (PFR), LV volumes throughout the cardiac cycle were calculated.

The tagged images were analyzed with the Harmonic Phase procedure [14]. Circumferential shortening was calculated over time during the cardiac cycle. For the LV free wall, septum, and RV free wall, the peak time (Tpeak) of circumferential shortening was calculated related to the ECG R-wave by automated routines [15].

### LV free wall, RV free wall, and septum definitions

The LV free wall was subdivided in 5 equal segments. The 2 segments of the LV wall that were in direct continuity with the septum were not included as part of the LV free wall. The RV free wall was delineated in the same way. The complete septum was taken for the calculation of the septal strain, from the anterior until the posterior connections with the ventricular wall. For the LV free wall, RV free wall, and septum, the strains and strain timing parameters were derived.

### RV End-Systolic Wall Stress

Our estimation of RV end-systolic (ES) wall stress for both the patients and control subjects starts from the law of Laplace [16]:

$$RV\;end-systolic\;wall\;stress=\frac{0.5\times \text{RV}\;\text{systolic}\;\text{pressure}\times \text{RV}\;\text{end-systolic}\;\text{radius}}{\text{RV}\;\text{end-systolic}\;\text{wall}\;\text{thickness}}$$

The systolic RV pressure is estimated by the *systolic *PAP. The RV ES radius of curvature is difficult to measure directly because of the RV's irregular shape. Therefore, we estimate this radius from the RVESV by assuming that this volume can be described by a sphere in both the patients and controls. Then the RV ES radius is:

$$End-systolic\;radius=0.620\times {\left(\text{RVESV}\right)}^{1/3}$$

The RV ES wall thickness is estimated by dividing the RV free wall ES volume by the RV free wall ES surface area. The RV free wall ES volume is obtained by contouring the RV free wall on every short-axis ES slice, and then applying Simpson's rule. The total RV ES surface area was calculated as: 4·*π *·radius^2^, with radius from the above equation. The RV free wall fraction of total RV surface is estimated as 2/3 part. Thus the RV free wall ES surface area is calculated as 2/3 times the total RV ES surface area.

### Statistics

GraphPad Prism version 4.0 (GraphPad Software, San Diego, California) was used for statistical calculations. All data was tested for normal distribution. We performed a 2-tailed paired Student *t *test to compare pre- and postoperative CMR measurements and hemodynamic measurements. The relations between the L-R delay in T_peak _versus LV stroke volume, LV PFR, six minute walking distance and RV wall stress were tested by linear regression. All data are described as mean ± standard deviation. A p value of < 0.05 was considered statistically significant

## Results

### Patients Characteristics

Patient characteristics are shown in Table 1. The median age of the patient population was 63 years (range 45-83) and 46% were female. The ECG-QRS width before PEA was 96 ± 9 ms. On the basis of ECG morphology, right bundle branch block was present in 1 patient. Coronary artery disease was not present in any of the patients studied. PEA was successful in all patients, resulting in a significant reduction in mean PAP (45 ± 12 vs 25 ± 6 mmHg; *P *< 0.001) and TPR (870 ± 391 vs 406 ± 171 dyn·s/cm^5^; *P *< 0.001). Invasive hemodynamic measurements are summarized in Table 2. The six-minute walk distance increased from 409 ± 109 m to 510 ± 91 m (*P *< 0.001).

**Table 1:** Patients Characteristics before PEA

Patient	Sex	Age (years)	HR (beats/min)	BP *s/d *(mmHg)	PAP *s/d/m *(mmHg)	PCWP (mmHg)	CO (l/min)	QRS *Width (ms)*	NYHA	Medication
**1**	m	78	74	120/70	80/30/47	5	3.7	104	3	ERA
**2**	f	50	74	120/80	110/30/56	13	2.7	108	3	none
**3**	f	58	75	115/75	83/37/54	14	3.6	108	3	ERA
**4**	f	43	72	120/80	78/24/45	15	4.2	88	3	ERA
**5**	m	66	49	150/85	55/14/29	11	5.8	92	2	ERA
**6**	m	69	75	130/80	42/14/26	9	5.9	94	2	ERA
**7**	F	68	88	110/70	109/33/58	5	2.3	88	4	ERA
**8**	f	61	87	95/65	78/33/50	6	5.5	92	3	ERA
**9**	m	51	78	175/85	72/29/45	8	4.8	108	3	ERA
**10**	f	60	69	131/90	60/47/53	12	4.0	82	3	ERA
**11**	f	51	76	120/75	48/17/26	10	3.7	86	3	ERA
**12**	m	74	80	160/90	70/49/59	13	3.7	102	3	ERA
**13**	m	55	79	130/80	55/18/32	12	5.2	96	2	ERA

BP = blood pressure; CO = cardiac output; CTEPH = Chronic Thrombo-Embolic Pulmonary Hypertension; ERA = endothelin receptor antagonist; HR = heart rate; inc. = incomplete; NYHA = New York Heart Association; PAP = pulmonary arterial pressure; PCWP = pulmonary capillary wedge pressure; RBBB = right bundle branch block; s/d/m = systolic/diastolic/mean.

**Table 2:** Invasive Hemodynamic data pre and post PEA

Parameter	Pre PEA	Post PEA ICU	p-Value
Heart Rate	75 ± 10	74 ± 8	**ns**
PAPsystolic (mm Hg)	72 ± 21	39 ± 14	**< 0.001**
PAPdiastolic (mm Hg)	28 ± 17	11 ± 5	**0.003**
PAPmean (mm Hg)	45 ± 12	25 ± 6	**< 0.001**
PVR(dyne s/cm5)	661 ± 338	n.m.	**-**
TPR (dyne s/cm5)	870 ± 391	406 ± 171	**0.001**
Cardiac output (l/min)	4.2 ± 1.1	4.8 ± 0.8	**ns**
BPsystolic (mm Hg)	120 ± 39	n.m.	**-**
BPsystolic (mm Hg)	78 ± 8	n.m.	**-**
PCWP (mm Hg)	7 ± 5	n.m.	**-**
RAP (mm Hg)	10 ± 3	n.m.	**-**

The 'n.m." indicates that the value is not measured on the intensive care unit (ICU). PAP = pulmonary artery pressure; PVR = pulmonary vascular resistance; RAP = right atrial pressure; other abbreviations as in Table 1

### CMR-derived ventricular volumes and function

As shown in Table 3, after PEA, there was a significant reduction in RVEDV and RVESV at 6 months after PEA. RV stroke volume tended to increase and RVEF increased. The preoperative hypertrophy of the RV decreased after PEA. Left ventricular EDV increased significantly with a stable LVESV. Therefore LV stroke volume improved significantly. In addition, LV peak filling rate (PFR) as corrected for LV-EDV increased significantly.

**Table 3:** Results of CMR volumetric parameters before and after PEA

CMR variables	Pre PEA	Post PEA	Mean Change (Postop to Preop)	p-Value
***Left Ventricle***				
end diastolic volume (ml)	98 ± 15	111 ± 19	13 ± 11	< 0.001
end systolic volume (ml)	28 ± 47	31 ± 46	1 ± 15	0.89
stroke volume (ml)	59 ± 13	72 ± 10	13 ± 11	< 0.001
ejection fraction (%)	62 ± 13	67 ± 5	5 ± 15	0.24
PFR (ml/s)	309 ± 89	474 ± 172	165 ± 150	0.002
PFR/end diastolic volume (s^-1^)	2.9 ± 0.8	4.2 ± 1.3	1.3 ± 1.1	0.003
***Right Ventricle***				
end diastolic volume (ml)	173 ± 38	125 ± 18	-47 ± 41	0.001
end systolic volume (ml)	107 ± 34	46 ± 16	-61 ± 31	< 0.001
stroke volume (ml)	65 ± 19	78 ± 14	13 ± 22	0.07
ejection fraction (%)	39 ± 12	63 ± 10	24 ± 14	< 0.001
Mass (g)	75 ± 19	51 ± 14	-24 ± 13	< 0.001

PFR = peak filling rate

### Images and strains

**Figure 1:**
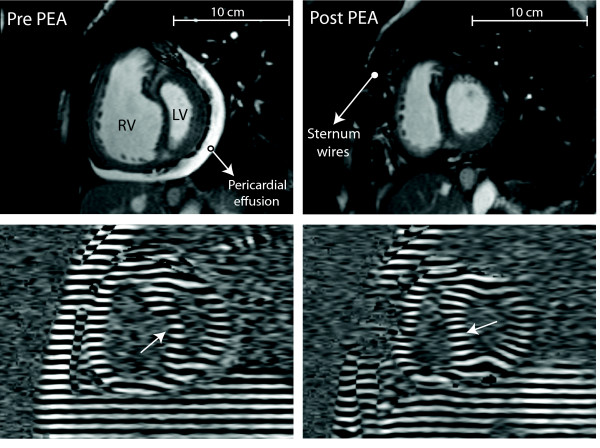
**Short-axis images **(top panels) **and short-axis tagged images **(bottom panels)**, at the time of peak right ventricular (RV) shortening in a patient with chronic thromboembolic pulmonary hypertension before and after pulmonary endarterectomy (PEA)**. Leftward ventricular septal bowing, as present before PEA, recovers 6 months after PEA (**white arrows**). CMR = cardiovascular magnetic resonance. RV = right ventricle, LV = left ventricle.

**Figure 2:**
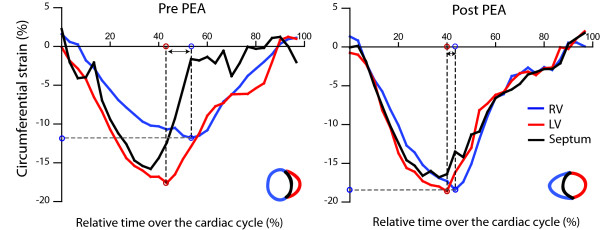
**Circumferential strain curves over time after the electrocardiographic R-wave for the left ventricular (LV) and right ventricular (RV) free walls and the septum for 1 patient pre (**left**) and post (**right**) PEA**. Pre PEA, the LV, RV, and septum start simultaneously with shortening (negative strain), but the RV reaches its peak later than the LV and the RV peak strain is lower. Post PEA, the L-R synchrony and RV peak strain have recovered.

Figure 1 shows short-axis cine images and short-axis tagged images at the time of RV peak strain before and after PEA. Figure 2 shows an example of the circumferential shortening curves during the cardiac cycle for the LV and RV free walls and the septum before and after PEA in one patient. Pre PEA, the LV and RV start simultaneously, but the RV reaches its peak later than the LV. Post operatively, the RV peak is not later than the LV peak. In the patients before PEA, RV free wall peak circumferential shortening was decreased (pre PEA, -13 ± 3% vs. control,-18 ± 2%: *P *< 0.001), while peak circumferential shortening of LV free wall did not differ as compared with the healthy controls. After PEA, RV peak circumferential shortening increased to normal values. (Figure 2).

### Timing parameters

**Figure 3:**
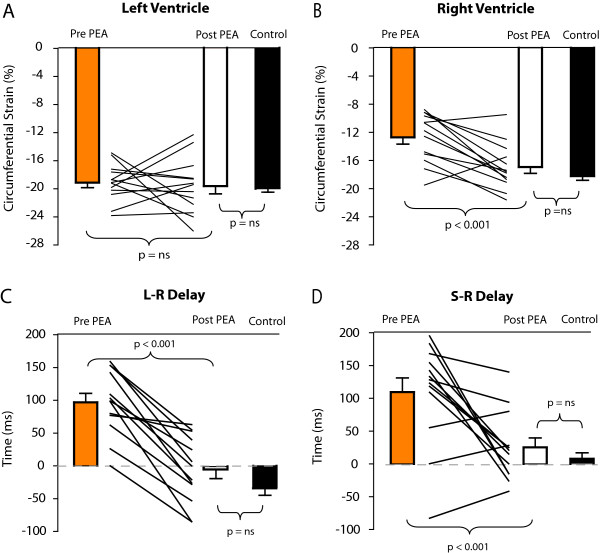
**Peak circumferential strain for the left (**A**) and right (**B**) ventricle, before and after PEA in the 13 CTEPH patients and the 8 healthy subjects**. Bar plots show the mean and the standard error of the mean, before PEA (open bars), and after PEA (closed bars). The panels C and D show the L-R delay (**C**) and the S-R delay (**D**), before and after PEA.

The results of the timing parameters pre-and post PEA are shown in Figure 3 and Table 4. Before PEA, the time to peak RV strain (*T*_peak_RV) was significantly longer compared with *T*_peak_LV, resulting in a L-R delay in peak strain. Postoperatively, *T*_peak_RV was significantly reduced; whereas *T*_peak_LV did not change. Consequently, the L-R delay decreased from 97 ± 49 ms to values not different from the controls i.e., they reached normal values: -4 ± 51 ms (*P *< 0.001). In addition the Septal to RV delay also normalized post PEA. Individual RV segmental data of the peak strains are presented in Table 5.

**Table 4:** CMR Strain and Timing Parameters before and after PEA

Strain and timing	Pre PEA	Post PEA	Mean Change (Postop to Preop)	p-Value
LV peak strain (%)	-19 ± 3	-20 ± 4	-0.5 ± 5	.70
RV peak strain (%)	-13 ± 3	-17 ± 3	-4 ± 3	0.001
SP peak strain (%)	-14 ± 3	-16 ± 4	-2 ± 3	0.09
RR (ms)	823 ± 70	840 ± 90	17 ± 134	0.64
T_max _LVSB (ms)	397 ± 77	Not observed	-	-
T_peak_RV (ms)	405 ± 61	352 ± 67	-53 ± 80	0.02
T_peak_LV (ms)	310 ± 46	356 ± 45	46 ± 80	0.09
T_peak_SP (ms)	296 ± 43	320 ± 58	-23 ± 82	0.33
LV to RV delay in T_peak _(ms)	97 ± 49	-4 ± 51	-101 ± 49	< 0.001
SP to RV delay in T_peak _(ms)	110 ± 78	25 ± 51	-85 ± 84	0.004

LV = left ventricular; LVSB = leftward septal bowing; RV = right ventricular; RR = R to R interval; SP = septum; T_peak _= Time to peak; T_max _= Time of maximal LVSB

**Table 5:** Individual segmental data of peak strain (in % circumferential shortening) for RV free wall segments anterior, mid and posterior

Patient	RV anterior segment			RV mid segment			RV posterior segment		
	**Pre**	**Post**	**Delta**	**Pre**	**Post**	**Delta**	**Pre**	**Post**	**Delta**

**1**	-5.7	-13.1	-7.4	-8.3	-13.3	-5.0	12.7	-13.4	-0.7
**2**	-11.0	-10.2	0.8	-10.1	-10.1	0.0	-13.2	-8.8	4.4
**3**	-4.7	-16.5	-11.7	-9.7	-11.0	-1.3	-11.1	-13.9	-2.8
**4**	-6.6	-18.9	-12.3	-8.5	-18.7	-10.2	-4.4	-17.0	-12.6
**5**	-15.4	-26.0	-10.6	-12.2	-21.0	-8.8	-12.4	-18.9	-6.5
**6**	-15.9	-19.1	-3.2	-11.8	-18.0	-6.2	-13.0	-19.9	-6.9
**7**	-16.2	-17.8	-1.6	-13.8	-17.4	-3.6	-16.4	-16.6	-0.2
**8**	-12.2	-17.8	-5.6	-11.1	-20.6	-9.5	-12.1	-16.8	-4.7
**9**	-11.3	-17.3	-6.0	-12.6	-18.2	-5.6	-10.7	-13.7	-3.0
**10**	-10.1	-15.2	-5.1	-10.0	-17.2	-7.2	-11.5	-18.0	-6.5
**11**	-11.3	-19.3	-8.0	-10.9	-21.9	-11	-17.0	-21.4	-4.4
**12**	-11.0	-15.4	-4.4	-8.6	-15.3	-6.7	-9.8	-13.2	-3.4
**13**	-18.3	-15.1	3.2	-18.8	-14.9	3.9	-21.4	-16.3	5.1

### Correlation and linear regression analysis

**Figure 4:**
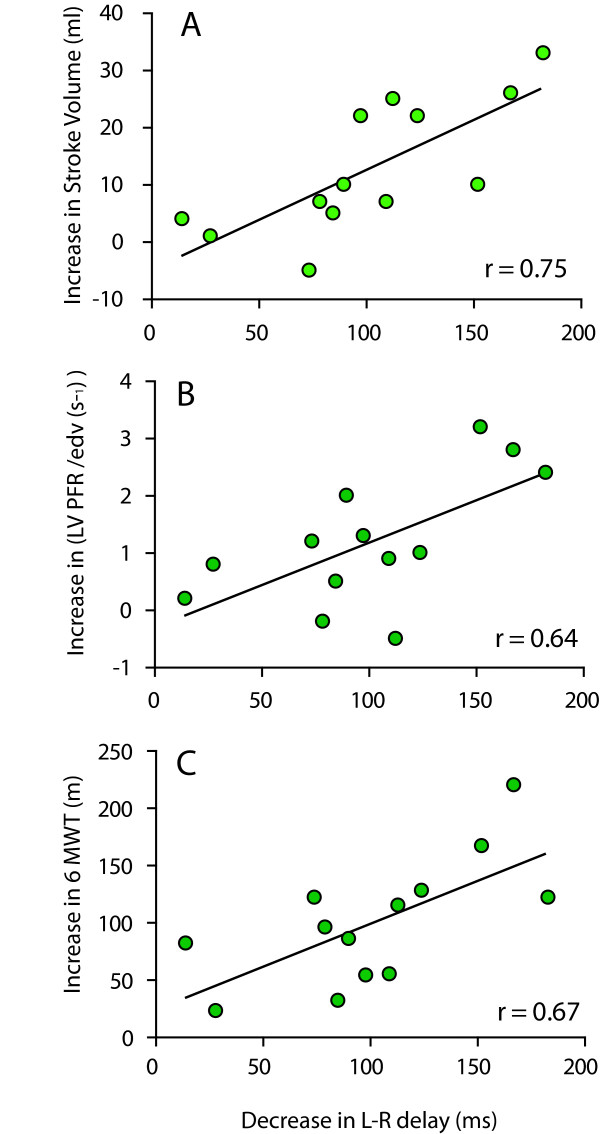
**Decrease in L-R delay versus (**A**) Increase in stroke volume; (**B**) increase in left ventricular peak filling rate (LV PFR) and (**C**) increase in six minute walking test**.

All variables satisfied the condition of normal distribution. The results of correlation and linear regression analysis are shown in Figure 4. Several recovery parameters were significantly associated with the reduction in L-R delay: increase in LV stroke volume (r = 0.75, *P *< 0.001), increase in normalized LV peak filling rate (r = 0.64, *P *< 0.001), and increase in 6 minute walking distance (r = 0.67, *P *< 0.001). In contrast, there was no significant correlation between the decrease in systolic pulmonary artery pressure and the decrease in L-R delay (r = 0.53, *P *= 0.07). Also there was no relation between the decrease in RV-radius and decrease in L-R delay (r = 0.37, *P *= 0.21), and no relation between the increase in end-systolic wall thickness and decrease in L-R delay (r = 0.19, *P *= 0.53).

### RV end-systolic wall stress

**Figure 5:**
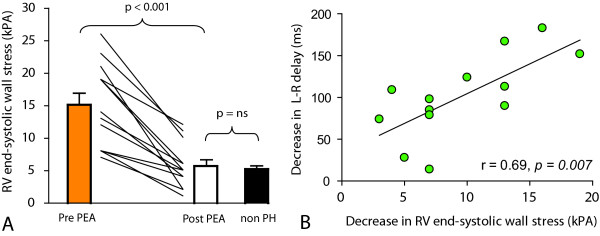
**RV end-systolic wall stress (**A**) before and after PEA in the 13 CTEPH patients and the 8 patients with normal right-sided pressures**. Bar plots show the mean and the standard error of the mean, before PEA (open bars), and after PEA (closed bars). (**B**) Linear regression between the decrease in left-to-right (L-R) delay in time to peak of circumferential shortening as dependent variable and the decrease in right ventricular (RV) end-systolic wall stress.

After PEA, the estimated RV end-systolic wall stress decreased significantly from 15.2 ± 6.4 kPa to 5.7 ± 3.4 kPa (*P *< 0.001), which was not different from the normal reference values of 5.3 ± 1.39 kPa (*P *= 0.78) (Figure 5a). In addition the RV end-systolic free wall thickness increased significantly after PEA (0.98 ± 0.17 cm vs. 1.21. ± 0.38 cm, *P *= 0.01). Furthermore, as shown in Figure 5b, the change in L-R delay correlated significantly with the reduction in RV end-systolic wall stress (r = 0.69, P = 0.007).

## Discussion

The major findings of the present study are: (1) after PEA, both the L-R delay in peak strains and estimates of end-systolic wall stress reverse to normal values; and (2) the change in L-R delay is predominantly associated with the change in RV end-systolic wall stress.

Our findings of a decrease in L-R dyssynchrony after unloading the RV, agree with the previous observations by Lurz et al [12] in patients where an RV-pulmonary artery conduit obstruction was relieved. Similar to our study, they reported that the reduction in RV wall stress correlated with the reduction in L-R delay. We showed that this also applies for patients with pulmonary hypertension and in addition we proved that the synchrony-recovery is mainly explained by the combined effect of reduction in PAP pressure, end-systolic radius and increased wall thickness. Since we also included healthy subjects, we were able to prove that RV peak strain, wall stress, as well as L-R synchrony reversed to normal values (Figure 3 and 5). This implies that the failing RV is capable of functional recovery after an intervention that reduces RV wall stress.

In this study, we performed the CMR measurements at least six months post-operatively, since previous reports showed that unloading the ventricle results in morphological and functional improvement over a period of at least 3 months [17,18]. In addition, Reesink et al [19] showed, by performing CMR after at least 4 months, that an almost complete reverse RV remodelling had taken place. However for the invasive pressures, we used the direct post-operative pressure measurements for the calculation of wall stress. The combination of post-operative CMR after 6 months and the postoperative pressure values is a limitation in this study, since the CMR derived values reflect a reverse-remodeled state whereas the post-PEA hemodynamics do not. Nevertheless, previous reports indicate that the pulmonary artery pressure remains stable during follow-up after PEA [20,21].

### Experimental Data

Our results agree with data on cardiac muscle. Brutsaert et al [22] and others [23] investigated the relationship between load and contraction duration by showing that an acute higher afterload imposed on isolated muscles increased the duration of contraction. Recently, Handoko et al [11] used an isolated langendorff-perfused heart from a chronic PH rat model and showed that ventricular dyssynchrony in peak pressure was induced by increasing RV volume and pressure, using an inflatable ventricular balloon. Thus the increase of myocardial wall stress leads to prolonged contraction duration, in experimental conditions as well.

### End-systolic wall stress in Pulmonary Hypertension

The finding that end-systolic wall stress is increased in CTEPH, is in accordance with earlier reports on pulmonary hypertension [24,25]. The increased wall stress does not only affect RV contraction duration, but also negatively affects myocardial perfusion [26] and subsequent glucose metabolism [25], and increases oxygen demand [27] Furthermore, Grossman et al [28] showed that increased wall stress induced dilatation, which in turn leads to further increase in wall stress and thereby a vicious circle of positive feedback is maintained.

Lowering end-systolic wall stress can be achieved by both a reduction of RV pressure and by an improvement in RV adaptation through concentric hypertrophy [29]. The relevance of this adaptation is manifest in patients with PH due to congenital heart disease: In these patients, the RV is more capable to cope with the increased afterload, probably because the RV has had more time to adapt by developing compensatory hypertrophy. This underscores the relevance of hypertrophy for lowering wall stress, and thereby for reducing L-R dyssynchrony in RV pressure overload.

### Limitation

The number of patients in this study was small. This is a consequence of the very invasive surgical PEA procedure, which can only be applied in a small subset of CTEPH patients. However, all statistical analyses were performed within patient, comparing the data before and after surgery by paired samples t-testing. This made it possible to obtain significant results in a small number.

Furthermore, a simplifying assumption is the description of the RV end-systolic volume by a spherical configuration, which is used in the calculation of RV wall stress for both the patients and the control subjects.

## Conclusions

In CTEPH patients, the L-R dyssynchrony in peak strain recovers to normal values after PEA. The RV end-systolic wall stress plays a key role in this recovery, reflecting a complex interplay of pulmonary artery pressure, RV radius and wall thickness.

## Competing interests

The authors declare that they have no competing interests.

## Authors' contributions

GM is responsible for conception and design of this study, data acquisition, analysis and interpretation of the results and drafting of the manuscript; AV is responsible for conception and design of this study and revising of the manuscript; TK carried out data acquisition, analysis and interpretation of the results. SS carried out data analysis; JK performed the surgical procedure and revising of the manuscript; PB revising of the manuscript; NS carried out data acquisition; JB carried out data acquisition, analysis and interpretation of the results; NW: is responsible for conception and design of this study; JM is responsible for conception and design of this study, interpretation of the results and drafting of the manuscript.
